# Florid Cemento‐Osseous Dysplasia Associated With Simple Bone Cysts: Radiographic Evolution Over 13 Years—A Case Report

**DOI:** 10.1155/crid/7764551

**Published:** 2026-11-09

**Authors:** Fabiana da Silva de Oliveira, Vinícius Teixeira Silva, Luana Rodrigues Santos, Adriane Bandeira Ramos de Lima, Norberto Nobuo Sugaya, Celso Augusto Lemos Júnior

**Affiliations:** ^1^ Department of Stomatology School of Dentistry University of São Paulo (FOUSP) São Paulo São Paulo Brazil usp.br

**Keywords:** case report, differential diagnosis, fibro-osseous lesions, florid cemento-osseous dysplasia, simple bone cyst

## Abstract

Florid cemento‐osseous dysplasia (FCOD) is a benign fibro‐osseous condition, typically asymptomatic and readily diagnosed because of its characteristic radiographic features, but diagnostic difficulties may arise when it is associated with secondary lesions. This report describes a rare case of FCOD associated with multiple simple bone cysts (SBCs), with radiographic evolution retrospectively documented over 13 years. A 45‐year‐old woman presented with multifocal mandibular lesions that were initially predominantly radiolucent and showed dynamic changes over time, including qualitative changes in radiographic appearance, progression to a mixed radiolucent–radiopaque pattern, and development of multiple bilateral intraosseous cavities. Episodes of pain were reported. Cone‐beam computed tomography demonstrated multiple intraosseous cavities, and surgical exploration of the right posterior lesion revealed an empty bone cavity, supporting the diagnosis of an SBC in this region. At the 2‐month postoperative follow‐up, the patient reported symptomatic improvement, and panoramic radiography showed early evidence of bone remodeling in the surgically explored area. This case demonstrates the importance of careful evaluation of radiolucent lesions in the context of FCOD to reduce the risk of potentially inappropriate interventions when radiolucent findings are interpreted in isolation. Integration of clinical findings, pulp vitality testing, and cone‐beam computed tomography is essential for accurate diagnosis, whereas analysis of serial radiographic examinations over time remains important given the evolving nature of these lesions.

## 1. Introduction

Cemento‐osseous dysplasia (COD) is a benign, nonneoplastic fibro‐osseous lesion that affects tooth‐bearing or previously tooth‐bearing regions of the jaws and is frequently detected incidentally on routine radiographic examinations. Its etiology remains uncertain. Radiographically, COD may present as a mixed radiolucent–radiopaque lesion or show predominantly radiopaque areas around tooth roots [1].

Histopathologically, COD is characterized by replacement of normal bone with fibrous connective tissue containing variable amounts of mineralized material, including immature bone and cementum‐like deposits [2]. Although its pathogenesis is not fully understood, it has been associated with alterations in bone remodeling, potentially influenced by local and hormonal factors [3].

COD is typically asymptomatic and occurs predominantly in middle‐aged women, with a higher prevalence among individuals of African ancestry and a predilection for the posterior mandible. The affected tissue tends to be densely mineralized and hypovascular, compromising its reparative capacity and increasing susceptibility to secondary infection [4].

Clinically, COD is classified into three subtypes—periapical, focal, and florid—each considered a distinct presentation within the same disease spectrum [5]. The florid form frequently involves bilateral regions of the mandible or both jaws and may become symptomatic at advanced stages, particularly in the presence of secondary infection [6, 7]. The lesion generally progresses from an initial radiolucent stage to a mixed pattern and, ultimately, to a predominantly radiopaque appearance as it matures [8].

Infectious complications may occur, especially after tooth extraction, periodontal disease, or trauma associated with prosthetic appliances or implants, favoring microbial contamination. In such cases, patients may present with pain, swelling, and purulent drainage. Management is generally conservative, with emphasis on clinical and radiographic follow‐up and avoidance of unnecessary invasive procedures [9].

The association of COD with other intraosseous lesions, particularly simple bone cysts (SBCs), warrants attention. This condition may manifest as empty bone cavities adjacent to dysplastic areas, which occasionally become symptomatic and may require surgical intervention to promote bone regeneration [10].

An SBC is an intraosseous cavity without an epithelial lining, generally asymptomatic and most often identified incidentally in the mandible [11]. Radiographically, it appears as a well‐defined osteolytic lesion that may mimic other odontogenic conditions [12]. The cortical plates are typically thin, and root resorption or tooth displacement is uncommon. Although rare, the association between SBC and COD may occur [13].

This association has been attributed to disturbances in bone remodeling and impaired vascularization, which may lead to venous obstruction, interstitial fluid accumulation, and formation of intraosseous cavities. Additional mechanisms may include intramedullary hemorrhage and subsequent organization of blood clots [14, 15]. The coexistence of SBC and COD, combined with the intrinsic variability of COD, may significantly alter the clinical and radiographic presentation, making differential diagnosis more challenging [16, 17].

Although the association between florid cemento‐osseous dysplasia (FCOD) and SBC has been reported, cases demonstrating multiple intraosseous cavities with dynamic long‐term evolution remain rare. Therefore, the present study reports a case with retrospective radiographic evolution over 13 years, emphasizing its diagnostic challenges and therapeutic implications.

## 2. Case Presentation

A 45‐year‐old woman presented to our service in 2026 with pain and swelling in the right posterior mandible. Her medical history was relevant only for controlled systemic arterial hypertension, treated with losartan 25 mg in the morning. As part of the diagnostic investigation, previous imaging examinations were retrieved, including a panoramic radiograph from 2013 that already demonstrated initial mandibular changes.

At that time (2013), the radiolucent areas were interpreted as lesions of endodontic origin, and no diagnosis of cemento‐osseous dysplasia or SBC was established.

The retrospectively reconstructed clinical and radiographic evolution between 2013 and 2026, as well as the interventions performed during this period, is summarized in Table 1.

**Table 1:** Clinical, radiographic, and therapeutic timeline of the case (2013–2026).

Year (age)	Clinical presentation	Radiographic evolution	Management and outcome
2013 (32 years**)**	Limited historical clinical information.	Multifocal predominantly radiolucent mandibular areas, retrospectively compatible with an early stage of FCOD. SBC could not be confirmed on panoramic radiography alone.	Endodontic treatment of Teeth 44 and 45 and extraction of Tooth 46 were performed after the initial interpretation of disease of endodontic origin.
2020 (39 years**)**	Return after approximately 7 years without follow‐up; episode of pain.	The radiolucent changes appeared qualitatively less evident compared with 2013. It was not possible to distinguish dysplastic changes from an associated SBC on panoramic radiography alone.	Endodontic treatment of Teeth 47 and 48 was performed; treatment of Tooth 37 was recommended under a provisional diagnosis of periapical granuloma.
2021 (40 years**)**	No new relevant clinical event was documented.	Radiographic appearance qualitatively similar to that in 2020.	No intervention related to the lesions was documented.
2022 (41 years**)**	Pain in the left posterior mandible.	Heterogeneous radiolucent–radiopaque pattern, retrospectively compatible with FCOD maturation.	Tooth 37 was extracted.
2023 (42 years**)**	Recurrent pain in the right posterior region.	More conspicuous radiolucent change with a cavitary appearance, retrospectively considered compatible with an SBC associated with FCOD.	Antibiotic therapy was prescribed, with temporary symptomatic improvement.
2026 (45 years**)**	Pain and swelling in the right posterior mandible.	CBCT demonstrated bilateral mixed‐pattern changes associated with FCOD and multiple intraosseous cavities.	Needle aspiration with an 18‐gauge needle yielded no content. Surgical exploration of the right posterior cavity revealed an empty bone cavity; curettage was performed.
2026 – 2 months postoperatively	Improvement in symptoms.	Early radiographic evidence of bone remodeling in the surgically explored area.	Clinical and radiographic follow‐up.

Subsequently, endodontic treatment of Teeth 44 and 45 and extraction of Tooth 46 were performed after the radiolucent findings had been interpreted as lesions of endodontic origin. The available historical records did not include documentation of pulp vitality testing or other clinical examinations that would allow retrospective assessment of the indications for these procedures.

Retrospective analysis of radiographic examinations obtained intermittently over a 13‐year period revealed that these findings corresponded to multifocal lesions, initially predominantly radiolucent, located in the posterior mandible and associated with subtle changes in the trabecular bone pattern. Over time, the lesions showed dynamic behavior, including qualitative changes in radiographic appearance and development of multiple intraosseous cavities in different mandibular regions (Figure 1).

**Figure 1:**
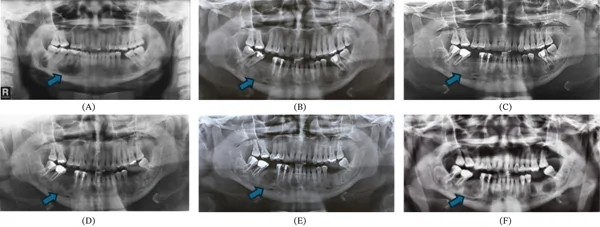
Sequence of panoramic radiographs showing the evolution of lesions compatible with florid cemento‐osseous dysplasia (FCOD) associated with simple bone cysts over time. Arrows indicate the right posterior region compared throughout the series; the additional arrow in Panel F highlights the left posterior cavity identified in 2026. (A) 2013, 32 years: Mildly radiolucent areas in the mandible, with poorly defined borders. (B) 2020, 39 years: The previously observed radiolucent areas appeared qualitatively less evident compared with the 2013 examination. (C) 2021, 40 years: Appearance qualitatively similar to the 2020 examination. (D) 2022, 41 years: Heterogeneous pattern, with radiolucent areas and radiopaque foci, compatible with FCOD maturation. (E) 2023, 42 years: More conspicuous radiolucent change with a cavitary appearance, retrospectively considered compatible with an SBC in the context of FCOD. (F) 2026, 45 years: A radiolucent area with a cavitary appearance in the region corresponding to the previously extracted Tooth 46 appears more conspicuous compared with previous examinations, in the context of the pain reported by the patient. In addition, other radiolucent areas with a cavitary appearance were identified in the left quadrant, reinforcing the dynamic and multifocal behavior of simple bone cysts associated with FCOD. *Note:* The evolution between 2013 and 2026 was retrospectively reconstructed from the available examinations and records; there was no continuous clinical follow‐up, including an interval of approximately 7 years without follow‐up. Comparisons among serial panoramic radiographs are qualitative because the examinations were not obtained in a standardized manner and no reproducible dimensional measurement method was applied. Historical records preceding the endodontic treatments and extractions do not document pulp vitality testing, periodontal status, caries/restorations, or objective signs of infection; therefore, these procedures cannot be retrospectively classified as unnecessary. The surgically explored right posterior cavity showed the characteristic finding of an empty bone cavity; the remaining cavities were considered radiographically compatible with SBCs, without individual surgical confirmation. FCOD, florid cemento‐osseous dysplasia; SBC, simple bone cyst; CBCT, cone‐beam computed tomography.

The patient remained without follow‐up for 7 years. In 2020, she returned with a new episode of pain in the right posterior mandible and underwent endodontic treatment of Teeth 47 and 48, together with a recommendation for treatment of Tooth 37 based on a provisional diagnosis of periapical granuloma. In 2022, after recurrent pain in the left posterior region, Tooth 37 was extracted.

Despite these interventions, pain recurred in 2023 in the right posterior region, including an edentulous area and a site adjacent to previously treated teeth. The radiographic examination demonstrated a more conspicuous radiolucent area with an intraosseous cavitary appearance, with temporary resolution of symptoms after antibiotic therapy.

Clinical examination in 2026 revealed no extraoral abnormalities. Intraorally, the mucosa was intact, with slight expansion of the buccal cortical plate on palpation in the right posterior mandible.

Cone‐beam computed tomography (CBCT) (Figure 2) demonstrated extensive bilateral mandibular involvement, characterized by mixed radiolucent–radiopaque changes and multiple predominantly well‐defined intraosseous cavities, unilocular in appearance, with thin margins and variable cortication. The right posterior cavity, corresponding to the edentulous region of Tooth 46, measured approximately 19 × 10 × 14 mm in the anteroposterior, buccolingual, and superoinferior axes, respectively. The largest left posterior cavity measured approximately 18 × 14–15 × 14 mm along the same axes. Both caused thinning of the buccal and lingual cortical plates, which remained continuous, without unequivocal evidence of cortical perforation. The inferior border of the mandible was preserved bilaterally. The mandibular canal remained identifiable inferior to the posterior cavities and was in close spatial relationship with them on some sections but without unequivocal interruption of its corticated boundaries. No significant root resorption or tooth displacement was observed.

**Figure 2:**
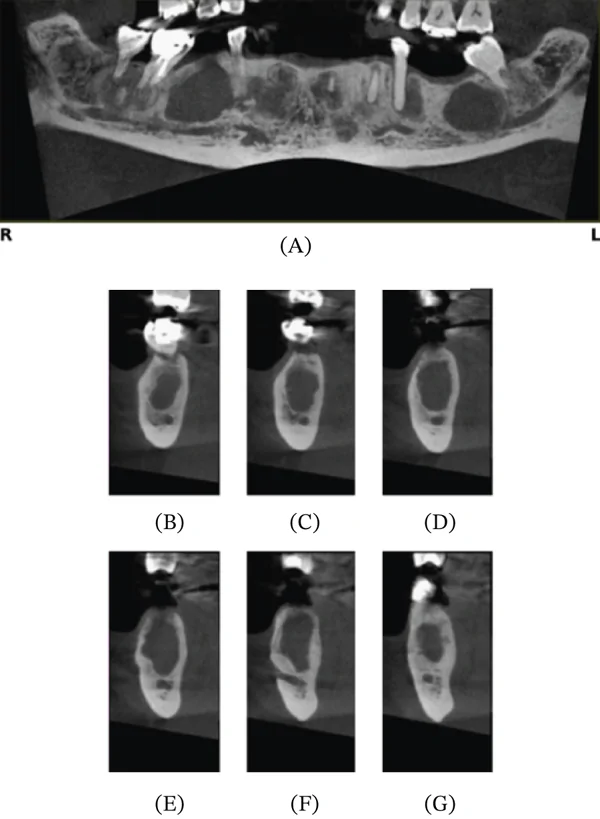
Cone‐beam computed tomography (CBCT) demonstrating multiple bilateral radiolucent intraosseous cavities in the mandible. (A) Panoramic reconstruction showing the bilateral posterior cavities. (B–G) Selected representative cross‐sectional images of the right posterior cavity, corresponding to the edentulous region of Tooth 46, demonstrating its buccolingual extent and thinning of the buccal and lingual cortical plates. The right posterior cavity measures approximately 19 × 10 × 14 mm in the anteroposterior, buccolingual, and superoinferior axes, respectively. The cortical plates are thinned but remain continuous, without unequivocal cortical perforation. The inferior mandibular border is preserved, and the mandibular canal remains identifiable inferior to the cavity, with close spatial relationship on some sections but without unequivocal interruption of its corticated boundaries.

Additionally, radiolucent areas were observed adjacent to the apices of Teeth 34 and 35, both of which showed a positive response to pulp vitality testing.

Given a more conspicuous radiolucent area with a cavitary appearance in the edentulous region corresponding to Tooth 46, associated with pain, needle aspiration was performed using an 18‐gauge needle; however, no fluid, blood, or other material was obtained from the cavity. The absence of aspirated content, together with persistent symptoms and the tomographic findings, led to the decision to proceed with surgical exploration for diagnostic clarification and management of the cavity. Subsequent surgical exploration revealed an empty bone cavity, and curettage was performed to promote bone regeneration (Figure 3). No significant soft‐tissue component suitable for histopathological examination was identified within the cavity; therefore, a representative tissue sample could not be obtained.

**Figure 3:**
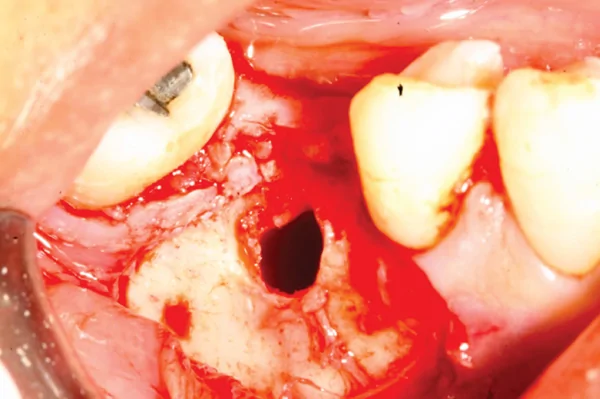
Intraoperative appearance after surgical access to the posterior mandible, demonstrating an empty bone cavity without fluid content, compatible with a simple bone cyst, located within an area of dysplastic bone.

At the 2‐month postoperative follow‐up, the patient reported improvement in symptoms. The panoramic radiograph demonstrated early radiographic evidence of bone remodeling within the explored cavity (Figure 4). The postoperative follow‐up available for this report was limited to this 2‐month period.

**Figure 4:**
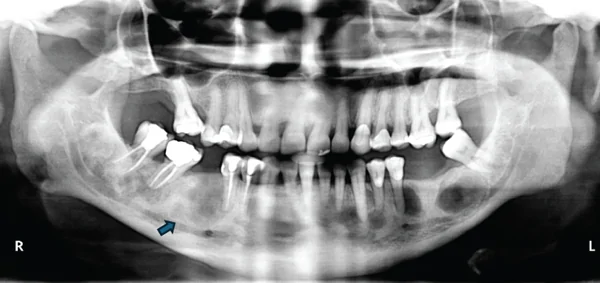
Follow‐up panoramic radiograph obtained 2 months after surgical exploration and curettage of the cavity in the right posterior mandible, demonstrating early radiographic evidence of bone remodeling in the explored area, with no evidence of postoperative complications. The arrow indicates the area of early bone remodeling.

Integration of the clinical and radiographic findings supported the diagnosis of FCOD, considering the multifocal and bilateral distribution of the mandibular changes, development of a mixed radiolucent–radiopaque pattern over time, and tomographic findings compatible with cemento‐osseous dysplasia. Additionally, the positive response to pulp vitality testing of Teeth 34 and 35 supported a nonendodontic origin for the corresponding radiolucencies.

Based on this set of findings, the diagnosis of FCOD was established using clinical and radiographic criteria, without biopsy of the dysplastic areas. Considering the characteristic radiographic presentation, multifocal and bilateral distribution, and absence of signs of aggressive behavior, tissue sampling from these areas was not considered necessary.

To distinguish FCOD maturation from the presence of cavities compatible with SBCs, the pattern and evolution of the imaging findings were considered. FCOD maturation was characterized by transition from initially radiolucent areas to a heterogeneous/mixed pattern with radiopaque foci, whereas areas interpreted as compatible with SBCs showed a more conspicuous cavitary radiolucent appearance and, on CBCT, predominantly unilocular, well‐defined morphology within areas of dysplastic bone. In the right posterior region, this interpretation was further supported by the absence of content on needle aspiration and the intraoperative identification of an empty bone cavity. The remaining cavities continued to be classified as radiographically compatible with SBCs associated with FCOD, without individual surgical confirmation.

## 3. Discussion

The main point of interest in this report is the uncommon association between FCOD and multiple bilateral SBCs, whose radiographic evolution could be retrospectively reconstructed over 13 years. Beyond the coexistence of the two conditions, the multiplicity of the cavities, their bilateral distribution, and the qualitative changes observed in examinations obtained at different time points illustrate the diagnostic complexity of this association and the risk of interpreting radiolucent areas in isolation.

At early stages, FCOD may be predominantly radiolucent and mimic inflammatory periapical changes [18, 19]. This diagnostic difficulty was reflected in the patient′s history, in which radiolucent areas had previously been interpreted as being of endodontic origin and led to endodontic treatments and extractions. Because the earlier records did not include pulp vitality testing, periodontal assessment, or sufficient additional clinical data, these interventions cannot be retrospectively classified as unnecessary. In the current evaluation, however, preserved pulp vitality of Teeth 34 and 35, the bilateral and multifocal distribution, and the surrounding mixed bone pattern favored a nonendodontic origin for the corresponding radiolucent findings.

The clinical presentation also reinforces the need for cautious interpretation. Although FCOD is frequently asymptomatic, pain and swelling are among the manifestations described in symptomatic cases, whereas clinically evident infection is less frequent [20–22]. For this patient, the available records documented recurrent episodes of pain but did not provide sufficient objective signs to confirm infection during the historical episodes. This distinction is relevant because it avoids retrospectively attributing an infectious mechanism to events that were not adequately documented.

Another central challenge was distinguishing radiographic maturation of FCOD from the appearance of cavities compatible with SBCs. On serial radiographs, the heterogeneous radiolucent–radiopaque pattern observed in 2022 was interpreted as compatible with FCOD maturation, whereas on subsequent examinations, more cavitary and conspicuous radiolucent areas raised the possibility of associated SBCs. Because the panoramic radiographs were obtained in a nonstandardized manner, these comparisons were considered qualitative. CBCT was fundamental for demonstrating the cavitary morphology, bilateral and multifocal distribution, cortical thinning, and relationship with adjacent anatomical structures, allowing more precise characterization of the changes [23, 24].

In the right posterior cavity, the absence of aspirated content, together with intraoperative identification of an empty bone cavity, supported the diagnosis of an SBC. No representative soft tissue was obtained for histopathological examination. Therefore, the diagnosis of SBC was more strongly supported only in this region; the remaining cavities were considered radiographically compatible with SBCs in the context of FCOD, without individual surgical confirmation. This distinction is important to avoid automatically interpreting every radiolucency observed within an area of FCOD as an SBC.

The association between FCOD and SBC is uncommon and may represent an additional challenge because radiolucent changes related to the dysplastic process and bone cavities may overlap on imaging [25]. Mechanisms involving local alterations in bone remodeling, reduced vascularization, and impaired lymphatic drainage have been proposed to explain this coexistence; however, these hypotheses remain speculative, and a direct causal relationship between FCOD progression and SBC development has not been established [26]. In this report, the occurrence of multiple bilateral cavities in dentate and edentulous regions, together with the possibility of reconstructing their appearance on examinations obtained between 2013 and 2026, distinguishes this presentation from many reports in which SBCs are described as isolated lesions or in small numbers.

The findings in this patient can also be contextualized using the pooled data from the meta‐analysis by Rave et al. [27], which included 51 patients with SBC associated with FCOD. The analyzed population showed a female predominance (92%) and a mean age of 41 years, characteristics close to those of the 45‐year‐old woman described here. Mandibular involvement was also predominant, with a pooled prevalence of 75.73%. By contrast, the multiplicity of cavities observed in this patient represents a less frequent presentation: 41 of 51 patients (80.4%) had a single SBC, whereas 10 (19.6%) had multiple SBCs. Surgical exploration was the most frequently reported diagnostic‐therapeutic approach, and curettage was performed in 83% of cases. Among patients with follow‐up, 52% showed complete healing and 32% partial healing. Recurrences or development of new lesions were also reported, with recurrence diagnosed after a mean of 6.6 years. These data underscore that the 2‐month postoperative follow‐up available in this report is insufficient to assess long‐term behavior.

Previous reports have also shown that SBCs associated with FCOD may exhibit atypical imaging features, including irregular borders on tomographic studies [28]. The temporal reconstruction was particularly useful for understanding lesion behavior but should be interpreted within its limitations. The examinations were obtained intermittently, there was an interval of approximately 7 years without follow‐up, and no reproducible method of dimensional measurement was used. Thus, the value of the radiographic series lies in demonstrating qualitative changes in appearance over time rather than objectively proving lesion growth or regression. Quantitative imaging methods, including CBCT texture analysis, have been investigated as auxiliary tools for differentiating cemento‐osseous dysplasia from inflammatory periapical changes, although they were not applied in this patient [29].

From a therapeutic standpoint, uncomplicated FCOD is generally managed conservatively, with clinical‐radiographic follow‐up and avoidance of invasive procedures whenever possible [30, 31].

The decision to surgically explore the right posterior cavity was driven by persistent symptoms, the cavitary appearance on CBCT, and the absence of aspirated content. Correlation among the clinical history, pulp vitality, serial radiographs, CBCT findings, and intraoperative findings was therefore essential for diagnostic reasoning and management planning.

A limitation of this report is the short available postoperative follow‐up period, limited to 2 months, which does not allow assessment of long‐term bone consolidation or evolution of the operated area.

## 4. Conclusion

This case highlights that isolated interpretation of radiographic findings in FCOD may contribute to diagnostic uncertainty and potentially inappropriate interventions. A comprehensive diagnostic approach integrating clinical examination, pulp vitality testing, and CBCT is essential for accurate diagnosis. Furthermore, comparative analysis of serial radiographic examinations over time may help identify the evolving changes in these lesions and support appropriate clinical decision‐making.

## 5. Patient Perspective

The patient reported improvement in her symptoms after the surgical procedure and expressed satisfaction with the clinical management and follow‐up.

## Author Contributions

Fabiana da Silva de Oliveira: Conceptualization, data curation, investigation, writing—original draft, and writing—review and editing. Vinícius Teixeira Silva: Data curation, investigation, and writing—review and editing. Luana Rodrigues Santos: Data curation, investigation, and writing—review and editing. Adriane Bandeira Ramos de Lima: Data curation, investigation, and writing—review and editing. Norberto Nobuo Sugaya: Supervision, validation, and writing—review and editing. Celso Augusto Lemos Júnior: Conceptualization, supervision, validation, and writing—review and editing.

## Funding

No funding was received for this manuscript.

## Ethics Statement

This manuscript describes a retrospective single‐patient case report based on routine clinical care. Formal ethics committee approval was not obtained for this case report. Written informed consent was obtained from the patient for publication of the clinical information and accompanying images, and the patient′s identity has been protected.

## Consent

Written informed consent was obtained from the patient for publication of this case report and any accompanying images. The patient′s identity has been protected.

## Conflicts of Interest

The authors declare no conflicts of interest.

## Supporting information


**Supporting Information** Additional supporting information can be found online in the Supporting Information section. File S1: CARE checklist.

## Data Availability

The data that support the findings of this study are available from the corresponding author upon reasonable request.

## References

[1] Zhang J. , Yu Y. , Tang W. , Pan J. , and Jing W. , Cemento-Osseous Dysplasia: A Detailed Comparison of the 2005 and 2017 WHO Classifications and Case Analysis, Cureus. (2023) 15, no. 11, e49041, 10.7759/cureus.49041.PMID. PMC10729712 38116337

[2] Vered M. and Wright J. M. , Update From the 5th Edition of the World Health Organization Classification of Head and Neck Tumors: Odontogenic and Maxillofacial Bone Tumours, Head and Neck Pathology. (2022) 16, no. 1, 63–75, 10.1007/s12105-021-01404-7.Epub. PMC9019005 35312978

[3] Nel C. , Yakoob Z. , Schouwstra C. M. , and van Heerden W. F. , Familial Florid Cemento-Osseous Dysplasia: A Report of Three Cases and Review of the Literature, Dentomaxillofacial Radiology. (2021) 50, no. 1, 20190486, 10.1259/dmfr.20190486.Epub. PMC7780834 32315206

[4] Jagtap R. , Gupta S. , Bhat M. , Mehta N. , and Gupta S. , Dilemma With Implant Placement in Patients With Florid Cemento-Osseous Dysplasia: A Literature Review, Science Progress. (2024) 107, no. 2, 368504241244657, 10.1177/00368504241244657.PMID. PMC11024584 38614470

[5] Urs A. B. , Augustine J. , and Gupta S. , Cemento-Osseous Dysplasia: Clinicopathological Spectrum of 10 Cases Analyzed in a Tertiary Dental Institute, Journal Of Oral And Maxillofacial Pathology. (2020) 24, no. 3, 10.4103/jomfp.JOMFP_139_20.Epub. PMC8083436 33967503

[6] Decani S. , Quatrale M. , Costa D. , Moneghini L. , and Varoni E. M. , Florid Cemento-Osseous Dysplasia: A Case Report and Review of Literature, Heliyon. (2024) 10, no. 14, e33746, 10.1016/j.heliyon.2024.e33746.PMID. PMC11305240 39114015

[7] Nishimura S. , Oda M. , Habu M. , Takahashi O. , Tsurushima H. , Otani T. , Yoshiga D. , Wakasugi-Sato N. , Matsumoto-Takeda S. , Nishina S. , Yoshii S. , Sasaguri M. , Yoshioka I. , and Morimoto Y. , Imaging Characteristics of Embedded Tooth-Associated Cemento-Osseous Dysplasia by Retrospective Study, Tomography. (2024) 10, no. 2, 231–242, 10.3390/tomography10020018.PMID. PMC10891647 38393286

[8] Demyati A. K. , Florid Cemento-Osseous Dysplasia Associated With Secondary Infection - A Case Report, Annals of Maxillofacial Surgery. (2023) 13, no. 2, 232–235, 10.4103/ams.ams_49_23.Epub. PMC10883215 38405575

[9] Alsufyani N. A. and Lam E. W. , Osseous (Cemento-Osseous) Dysplasia of the Jaws: Clinical and Radiographic Analysis, Journal of the Canadian Dental Association. (2011) 77, 21683027. 21683027

[10] Chinckr C. P. , Tinoco L. V. C. , Souza D. F. , and Pinheiro T. N. , Large Simple Bone Cyst With Florid Cemento-Osseous Dysplasia: A Case Report, Oral Surgery, Oral Medicine, Oral Pathology and Oral Radiology. (2020) 129, no. 1, 10.1016/j.oooo.2019.06.174.

[11] Lima L. B. , de Freitas Filho S. A. , Barbosa de Paulo L. F. , Servato J. P. , Rosa R. R. , Faria P. R. , Loyola A. M. , and Cardoso S. V. , Simple Bone Cyst: Description of 60 Cases Seen at a Brazilian School of Dentistry and Review of International Literature, Medicina Oral, Patologia Oral Y Cirugia Bucal. (2020) 25, no. 5, e616–e625, 10.4317/medoral.23638.PMID. PMC7473432 32683391

[12] Jiang Z. Y. , Lan T. J. , Cai W. X. , and Tao Q. , Primary Clinical Study of Radiomics for Diagnosing Simple Bone Cyst of the Jaw, Dentomaxillofacial Radiology. (2021) 50, no. 7, 20200384, 10.1259/dmfr.20200384.Epub. PMC8474131 34233493

[13] Tufan Yucesoy M. , Duman Tepe R. , Soluk Tekkesin M. , and Cakir K. H. , Radiographic and Histopathologic Characteristics of Simple Bone Cysts: A Retrospective Study of 46 Cases, BMC Oral Health. (2025) 26, no. 1, 10.1186/s12903-025-07511-z. PMC12822239 41402962

[14] Rao K. A. , Shetty S. R. , Babu S. G. , and Castelino R. L. , Co-Occurence of Florid Cemento-Osseous Dysplasia and Simple Bone Cyst: A Case Report, Journal Of Oral & Maxillofacial Research. (2011) 2, no. 3, 10.5037/jomr.2011.2305.PMID. PMC3886077 24421997

[15] Rollin M. and Taihi I. , Simple Bone Cyst Within Florid Cemento-Osseous Dysplasia: A Report of Two Cases, Cureus. (2024) 16, no. 7, e65803, 10.7759/cureus.65803.PMID. PMC11362631 39219876

[16] Hajjami F. , Ouertani H. , Brahem H. , Mehrez H. , Blouza I. , and Khattech M. B. , Association of Simple Bone Cyst and Cemento-Osseous Dysplasia: A Long-Term Follow-Up, Clinical Case Reports. (2023) 11, no. 4, e7161, 10.1002/ccr3.7161.PMID. PMC10062316 37006847

[17] Gumru B. , Akkitap M. P. , Deveci S. , and Idman E. , A Retrospective Cone Beam Computed Tomography Analysis of Cemento-Osseous Dysplasia, Journal of Dental Sciences. (2021) 16, no. 4, 1154–1161, 10.1016/j.jds.2021.03.009.Epub. PMC8403794 34484583

[18] Nam I. , Ryu J. , Shin S. H. , Kim Y. D. , and Lee J. Y. , Cemento-Osseous Dysplasia: Clinical Presentation and Symptoms, Journal of the Korean Association of Oral and Maxillofacial Surgeons. (2022) 48, no. 2, 79–84, 10.5125/jkaoms.2022.48.2.79.PMID. PMC9065647 35491138

[19] Daviet-Noual V. , Ejeil A. L. , Gossiome C. , Moreau N. , and Salmon B. , Differentiating Early Stage Florid Osseous Dysplasia From Periapical Endodontic Lesions: A Radiological-Based Diagnostic Algorithm, BMC Oral Health. (2017) 17, no. 1, 10.1186/s12903-017-0455-5.PMID. PMC5745995 29284472

[20] Benaessa M. M. , Mahomed F. , and Ngwenya S. P. , A Retrospective Clinico-Pathologic Analysis of Cemento-Osseous Dysplasia in a South African Patient Population, African Health Sciences. (2019) 19, no. 4, 3154–3159, 10.4314/ahs.v19i4.38.PMID. PMC7040344 32127892

[21] Kato C. N. A. O. , de Arruda J. A. A. , Mendes P. A. , Neiva I. M. , Abreu L. G. , Moreno A. , Silva T. A. , Souza L. N. , and Mesquita R. A. , Infected Cemento-Osseous Dysplasia: Analysis of 66 Cases and Literature Review, Head And Neck Pathology. (2020) 14, no. 1, 173–182, 10.1007/s12105-019-01037-x. PMC7021850 31011984

[22] Gabay M. , DiPede L. , Fornatora M. , Yang J. , and Ogwo C. , Treatment Indications for Symptomatic Versus Asymptomatic Florid Cemento-Osseous Dysplasia in Adult Patients: A Systematic Review, Oral Surgery, Oral Medicine, Oral Pathology and Oral Radiology. (2024) 138, no. 6, 686–701, 10.1016/j.oooo.2024.06.011.Epub. 39306516

[23] McLean A. C. and Vargas P. A. , Cystic Lesions of the Jaws: The Top 10 Differential Diagnoses to Ponder, Head And Neck Pathology. (2023) 17, no. 1, 85–98, 10.1007/s12105-023-01525-1.Epub. PMC10063769 36928736

[24] Nelke K. , Karpiński M. , Scharoch M. , Janeczek M. , Małyszek A. , Kalfarentzos E. , Mavrakos E. , Kuropka P. , Perisanidis C. , and Dobrzyński M. , A Cystic-Like Lesion of Uncertain Origin-A Discussion on Cemento-Osseous Dysplasia and Traumatic Bone Cysts, Diagnostics. (2025) 15, no. 18, 10.3390/diagnostics15182312.PMID. PMC12468121 41008683

[25] Lee Y. P. , Huang B. W. , Chiang C. P. , and Hwang M. J. , Florid Cemento-Osseous Dysplasia With Concomitant Occurrence of Two Simple Bone Cysts in the Mandible-Case Report, Journal of Dental Sciences. (2020) 15, no. 3, 383–385, 10.1016/j.jds.2020.05.009.Epub. PMC7486495 32952896

[26] Berberi A. , Clinical, CBCT and Histological Analysis of a Florid Cemento-Osseous Dysplasia With Co-Occurrence of Simple Bone Cyst in the Mandible: A Case Report, Journal of Dentistry. (2024) 25, no. 3, 278–287, 10.30476/dentjods.2024.101163.2276. PMC11452594 39371950

[27] Rave L. , Rollin M. , Porporatti A. L. , and Taïhi I. , Clinical Characteristics of Simple Bone Cyst Associated Within Florid Cemento-Osseous Dysplasia: A Meta-Analysis, Medicina Oral, Patología Oral y Cirugía Bucal. (2025) 30, no. 5, e709–e720, 10.4317/medoral.27205.PMID. PMC12395575 40818140

[28] Kojima I. , Nishioka T. , Sakamoto M. , Sai Y. , Ezoe Y. , Iikubo M. , Kumamoto H. , and Takahashi T. , Florid Cemento-Osseous Dysplasia-Associated Simple Bone Cyst Showing Marked Irregular Border and High Apparent Diffusion Coefficient Value, Case Reports in Dentistry. (2020) 22, no. 2020, 8854428, 10.1155/2020/8854428. PMC7528028 33029434

[29] Park S. , Jeon S. J. , Yeom H. G. , and Seo M. S. , Differential Diagnosis of Cemento-Osseous Dysplasia and Periapical Cyst Using Texture Analysis of CBCT, BMC Oral Health. (2024) 24, no. 1, 10.1186/s12903-024-04208-7.PMID. PMC11008037 38605361

[30] Fenerty S. , Shaw W. , Verma R. , Syed A. B. , Kuklani R. , Yang J. , and Ali S. , Florid Cemento-Osseous Dysplasia: Review of an Uncommon Fibro-Osseous Lesion of the Jaw With Important Clinical Implications, Skeletal Radiology. (2017) 46, no. 5, 581–590, 10.1007/s00256-017-2590-0.Epub. 28194495

[31] Mlouka M. , Tlili M. , Khanfir F. , Hamrouni A. , Khalfi M. S. , and Ben A. F. , Implant Placement in a Focal Cemento-Osseous Dysplasia: A Modified Protocol With a Successful Outcome, Clinical Case Reports. (2022) 10, no. 1, e05307, 10.1002/ccr3.5307.PMID. PMC8777049 35079398

