# Supplementary material for: Florid Cemento‐Osseous Dysplasia Associated With Simple Bone Cysts: Radiographic Evolution Over 13 Years—A Case Report
Source: Case Rep Dent. 2026 Nov 9;2026:7764551. doi: 10.1155/crid/7764551 (PMC13748942; doi:10.1155/crid/7764551)
Supplement: Supplementary file 1 [file CRID-2026-7764551-s001.docx]

**CARE CHECKLIST**

*Checklist of information to include when writing a case report (CARE 2013)*

**Manuscript:** FLORID CEMENTO-OSSEOUS DYSPLASIA ASSOCIATED WITH SIMPLE BONE CYSTS: RADIOGRAPHIC EVOLUTION OVER 13 YEARS — A CASE REPORT

**Note:** Page numbers refer to the current 17-page English manuscript submitted with this checklist.

| **Topic** | **Item** | **Checklist item description** | **Reported on page(s)** |
| --- | --- | --- | --- |
| Title | 1 | The diagnosis or intervention of primary focus followed by the words “case report”. | p. 1 |
| Key Words | 2 | 2 to 5 key words that identify diagnoses or interventions in this case report, including “case report”. | p. 2 |
| Abstract | 3a | Introduction: What is unique about this case and what does it add to the scientific literature? | p. 2 |
| Abstract | 3b | Main symptoms and/or important clinical findings. | p. 2 |
| Abstract | 3c | The main diagnoses, therapeutic interventions, and outcomes. | p. 2 |
| Abstract | 3d | Conclusion—What is the main “take-away” lesson(s) from this case? | p. 2 |
| Introduction | 4 | One or two paragraphs summarizing why this case is unique (may include references). | pp. 3–4 |
| Patient Information | 5a | De-identified patient-specific information. | p. 4 |
| Patient Information | 5b | Primary concerns and symptoms of the patient. | pp. 4–5 |
| Patient Information | 5c | Medical, family, and psychosocial history including relevant genetic information. | p. 4 (medical history reported; family/psychosocial/genetic history not reported) |
| Patient Information | 5d | Relevant past interventions with outcomes. | pp. 4–6 |
| Clinical Findings | 6 | Describe significant physical examination and important clinical findings. | p. 5 |
| Timeline | 7 | Historical and current information from this episode of care organized as a timeline. | p. 6 (Table 1) |
| Diagnostic Assessment | 8a | Diagnostic testing (such as physical examination, laboratory testing, imaging, surveys). | pp. 5, 7–10 |
| Diagnostic Assessment | 8b | Diagnostic challenges (such as access to testing, financial, or cultural). | pp. 4–5, 10–12 |
| Diagnostic Assessment | 8c | Diagnosis (including other diagnoses considered). | pp. 4–5, 10–12 |
| Diagnostic Assessment | 8d | Prognosis (such as staging in oncology) where applicable. | N/A |
| Therapeutic Intervention | 9a | Types of therapeutic intervention (such as pharmacologic, surgical, preventive, self-care). | pp. 5–6, 9 |
| Therapeutic Intervention | 9b | Administration of therapeutic intervention (such as dosage, strength, duration). | pp. 5–6, 9 (details available in the clinical record are reported) |
| Therapeutic Intervention | 9c | Changes in therapeutic intervention (with rationale). | pp. 5–6, 9 |
| Follow-up and Outcomes | 10a | Clinician- and patient-assessed outcomes (if available). | pp. 9, 13 |
| Follow-up and Outcomes | 10b | Important follow-up diagnostic and other test results. | pp. 9–10 |
| Follow-up and Outcomes | 10c | Intervention adherence and tolerability (How was this assessed?). | N/A for the surgical intervention; adherence/tolerability to prior therapies was not documented |
| Follow-up and Outcomes | 10d | Adverse and unanticipated events. | p. 10 (no postoperative complications reported) |
| Discussion | 11a | A scientific discussion of the strengths AND limitations associated with this case report. | pp. 11–13 |
| Discussion | 11b | Discussion of the relevant medical literature with references. | pp. 11–13 |
| Discussion | 11c | The scientific rationale for any conclusions (including assessment of possible causes). | pp. 11–13 |
| Discussion | 11d | The primary “take-away” lessons of this case report (without references) in a one-paragraph conclusion. | p. 13 |
| Patient Perspective | 12 | The patient should share their perspective in one to two paragraphs on the treatment(s) they received. | p. 13 |
| Informed Consent | 13 | Did the patient give informed consent? Please provide if requested. | Yes — p. 14 (Patient Consent) |

**Source:** CARE Checklist (2013), CARE Case Report Guidelines.
